# Effect of green propolis on oral epithelial dysplasia in rats

**DOI:** 10.1590/S1808-86942011000300002

**Published:** 2015-10-19

**Authors:** Danielle Rodrigues Ribeiro Cavalcante, Paula Santos de Oliveira, Sandro Mota Góis, Andréa Ferreira Soares, Juliana Cordeiro Cardoso, Francine Ferreira Padilha, Ricardo Luiz Cavalcanti de Albuquerque Júnior

**Affiliations:** 1 Master's degree student in Health and Environment, Tiradentes University, Aracaju/SE, Brazil; 2 Biology graduate, Tiradentes University, Aracaju/SE, Brazil. Teacher; 3 Biology graduate, Tiradentes University, Aracaju/SE, Brazil. Teacher; 4 Doctoral degree in Oral Pathology (UFRN). Adjunct professor at the Sergipe Federal University, Aracaju/SE, Brazil; 5 Doctoral degree in Pharmacy (USP). Full professor in the graduate program on Health and Environment, Tiradentes University, Aracaju/SE, Brazil; 6 Doctoral degree in Food Science (UNICAMP). Full professor in the graduate program on Health and Environment, Tiradentes University, Aracaju/SE, Brazil; 7 Doctoral degree in Oral Pathology (UFRN). Full professor in the graduate program on Health and Environment, Tiradentes University, Aracaju/SE, Brazil; Fundaçã o de Apoio a Pesquisa e Inovaçã o Tecnológica do Estado de Sergipe (FAPITEC)

**Keywords:** 9,10-Dimetil-1,2-benzanthracene, mouth neoplasms, propolis

## Abstract

Studies have demonstrated that flavonoid compounds of green propolis have antitumoral activity. **Study Design**: Experimental study.

**Aims:**

To evaluate the effect of a hydroalcoholic extract of green propolis (EPV) on chemically induced epithelial dysplasias in rat tongues.

**Methods and Materials:**

DMBA was brushed on the lingual dorsum of rats 3x/week on alternate days - 100 (PROP1), 200 (PROP2) and 300 mg/kg (PROP3) EPV was administered orally for 20 weeks. EPV or DMBA were replaced by their vehicles and applied as positive (TUM1 and TUM2) and negative controls (CTR1 and CTR2), respectively. The lingual epithelium was histologically analyzed and graded according a binary system and the WHO classification; the data were compared using ANOVA (*p<0.05).

**Results:**

The EPV yield was 41% and the flavonoid yield was 0.95±0.44%. According to the Binary System, TUM1, TUM2 and PROP1 were considered high risk lesions, with significantly higher morphological alteration rates compared to the other groups (*p*<0.05), which were considered low risk lesions. Based on the WHO classification, moderate dysplasia was TUM1 and TUM2, mild dysplasia was PROP1, PROP2 and PROP3, and non-dysplastic epithelium was CTR1 and CTR2.

**Conclusion:**

EPV seems to play an important protective role against chemically-induced lingual carcinogenesis in rats.

## INTRODUCTION

Oral cancer is a public health problem in Brazil. The Brazilian National Cancer Institute (Instituto Nacional do Cancer or INCA^1^) estimates that there were 10,330 new cases in men and 3,790 new cases in women per 100,000 inhabitants in 2010. The incidence is higher in white males aged over 40 years that are smokers and/or consumers of alcoholic beverages. The clinical presentation of the oral squamous cell carcinoma is preceded by premalignant lesions named oral leukoplakia.^2^, ^3^ These premalignant lesions may present a wide array of histological findings, such as hyperkeratosis, acanthosis, epithelial atrophy, different degrees of dysplasia, and a chronic inflammatory infiltrate in the underlying connective tissue.^3^ Epithelial dysplasia may be characterized by changes in epithelial renewal and maturation resulting in structural and cytological alterations.^3^, ^4^

The treatment for this condition varies and is closely related with the disease stage and the origin of the tumor. Surgery and radiotherapy may be combined or not with chemotherapy as supplementary therapy. Neck dissection is done if there are metastases to lymph nodes.^5^

Several studies are underway to propose new treatments, such as the use of phytotherapy to treat neoplasms. Khalil^5^ presented encouraging results in the in vitro treatment of human cancer cells and in animals by using chemical compounds similar to those found in propolis. Orsolic et al.^6^ also showed that several hydrosoluble compounds of propolis, such as caffeic acid, caffeic acid phenethyl ester, and quercetin, could be extremely useful for controlling tumor growth in experimental models. Luo et al.^7^ showed in a study of a compound isolated from Brazilian propolis - PM-3 (3-[2-dimethyl-8-(3-methyl-2-butenyl)benzopyran]-6-propenoic) acid - that inhibited significantly the growth of cancer MCF-7 cells from human breasts. This evaluation was associated with cell inhibition in the cell cycle and induction of apoptosis.

In the present study, the chemical carcinogen 9,10-dimethyl-1,2-benzanthracene (DMBA) was used; it behaves experimentally as an initiator and promoting carcinogenesis agent. Our intention was to assess the antitumor activity of different doses of a hydroalcoholic extract of green propolis over the initial stages of mouth cancer, using a histomorphological analysis of DMBA-induced oral lesions. Another purpose was to compare the applicability of two histological grading systems for oral epithelial dysplasia (WHO and Binary System) in experimental studies on the chemoprevention of carcinogenesis.

## MATERIAL AND METHODS

### Ethical perspectives

Ethical principles of the COBEA (Brazilian College for Animal Experimentation) for experiments in animals were applied in this study. The institutional review board approved the study (approval no. 191208). The study was carried out at the biotherium and the morphology laboratory of this institution.

### Gathering propolis

Propolis was gathered from apiaries in previously labeled Langstrot type boxes. The material was labeled and placed in sterile refrigerated containers and sent to the laboratory. The location, conditions of the swarm, blooms, climate during collection, and other pertinent information about the characteristics of the propolis were recorded during the collection work at the apiaries.

### Obtaining a propolis extract

The extract was obtained by using Park et al.'s^8^ method. One gram of propolis was obtained by grinding and homogenizing the sample and adding 100 mL of a 70% hydroalcoholic solution. Extraction was done by agitating at room temperature for 24 h. The sample was then filtered and the solvent was rotoevaporated. The resulting powder was stored in a sterile test tube with screw cap and stored refrigerated. The extraction yield relative to the initial mass of propolis was calculated and expressed as a percentage.

### Determining the concentration of flavonoids

The concentration of total flavonoids was established by Adelmann's^9^ method. For this, 15 to 1000 μL of the extract (concentrations of 5 to 100 mg/mL) were added to a solution containing 0.1 mL of 10% aluminum nitrate and 0.1 mL of potassium acetate (1 mol/liter). The end volume was completed to 5 mL with 80% ethanol. The samples were homogenized and absorbance was measured spectrophotometrically at 415 nm after 40 at room temperature. Quercetin at 5 to 50 μg/mL concentrations, dissolved in ethanol, was used to build the standard concentration curve; total flavonoid values were expressed as quercetin equivalents (mg of quercetin in 100 mg of total solids).

### Biological assay

The animals comprised 42 adult male rats (*Rattus novergicus albinus*, Wistar lineage) with a body mass of about 350 ±50g, originating from the biotherium of the institution. They were randomly allocated to seven experimental groups. The choice of animals was based on their nature, offering good handling and monitoring conditions (Frame 1).

**Frame 1:** Distribution of experimental and control groups

GROUPS	PRODUCT PAINTED ON THE RAT TONGUE	PRODUCT ADMINISTERED BY GAVAGEM
CTR1	Distilled water	Distilled water 1 mL + 2 drops of 2% tween 80
CTR2	Distilled water	Hydroalcoholic extract of green propolis - 100 mg/kg
TUM1	0.5% DMBA	Distilled water 3 mL
TUM2	0.5% DMBA	2% tween 80 3 mL
PROP1	0.5% DMBA	Hydroalcoholic extract of green propolis - 100 mg/kg
PROP2	0.5% DMBA	Hydroalcoholic extract of green propolis - 200 mg/kg
PROP3	0.5% DMBA	Hydroalcoholic extract of green propolis - 300 mg/kg

Animals were kept in cages with wood shavings bedding that was replaced daily; the temperature was controlled at 22°C, light was applied in a 12 h light/darkness scale, water was given ad libitum, and a standard diet with Labina® (Purina, Sao Paulo, Brazil) was given. After reaching the abovementioned weight, the animals underwent the procedures for inducing experimental chemical carcinogenesis at the biotherium of the institution.

### Procedure for chemically inducing carcinogenesis

Carcinogenesis was induced in the middle third of the dorsum of the tongue in the rats of groups TUM1, TUM2, PROP1, PROP2, and PROP3 by applying 9,10 dimethyl 1,2-benzantracene (DMBA, Sigma-Aldrich, St. Louis, USA) topically. One gram of the carcinogen was diluted in 200 mL of acetone (P.A.) to obtain a 0.5% solution. The induction process consisted of dipping a sable (n^o^ 0) brush in the solution, removing the excess, and painting the dorsum of the tongue of the animals. Painting was done twice for each induction process with the animal immobilized but not sedated. The carcinogen was applied on every other day during 20 weeks.^10^ Distilled water was painted on the tongue of animals in groups CTR1 and CTR2, using the same technical procedures as in the other groups.

### Gavage procedures

The dry extract was again placed in suspension at 2% Tween 80 at 10 mg/mL to administer the green propolis extract. An oral dose of green propolis hydroalcoholic extract (administered by gavage) was given to animals in groups PROP1 (100 mg/kg), PROP2 (200 mg/kg), and PROP3 (300 mg/kg). Distilled water was given to animals in groups CTR1 and CTR2; three milliliters of 2% tween 80 was given to groups TUM1 and TUM2. The negative control groups were CTR1 and CTR2, which were not given DMBA; the positive control groups were TUM1 and TUM2, which were given DMBA). These substances were administered orally every other day (differing from the days of DMBA application). Gavage was done during one week with the same dosages before inducing carcinogenesis, for 20 weeks, to verify possible adverse reactions to the natural product, as preconized by Kavitha & Manoharan (2006).^10^

### Procedures for the histomorphological analysis of specimens

After 20 weeks, animals were euthanized in a CO_2_ chamber for post-mortem removal of the painted area. Tissue specimens were fixed in buffered formaldehyde (10%, pH 7.4) for 24 h, dehydrated in increasing ethyl alcohol solutions, and diaphanized in xylol for inclusion in paraffin. Histological sections were 5μm thick, which were hematoxylin-eosin stained for analysis using a light microscope (Olympus CX31 optic microscope) by three trained observers.

Lesions were classified according to the histological grading systems proposed by the World Health Organization (WHO)^4^ and Kujan et al.'s binary system.^11^ The following architectural and cytologic features were assessed: **1) Architectural**: irregular epithelial stratification; loss of polarity of basal layer cells; droplet-shaped epithelial projections; increased number of mitotic figures; presence of abnormal mitotic figures in the upper half of the epithelial (high mitoses); premature keratinization in single cells and keratin pearls in epithelial projections; **2) Citologic**: abnormal variation in nucleus size; nuclear pleomorphism; abnormal variation in cell size; cell pleomorphism; increased nucleus/cytoplasm ratio; increased size of the nucleus; abnormal mitotic figures; increased number and size of nucleoli; nuclear hyperchromatism. According to the WHO,^4^ changes were classified into: mild dysplasia, if the abovementioned changes were restricted to the lower third of the epithelium (basal and parabasal layers); moderate dysplasia, when these changes reached the middle third of the epithelium (middle layers of the squamous layer), and severe dysplasia, when architectural and cytologic changes were located beyond the middle third of the epithelium. In the binary system,^11^ epithelial changes were categorized as: 1) high-risk lesions - presence of four or more architectural alterations and/or five or more cytologic changes; and 2) low-risk lesions - presence of less than four architectural changes and less than five cytologic alterations.

The mean values of the final scores were compared among groups, by applying analysis of variance (ANOVA) and Tukey's post hoc test. The differences among means were significant if *p* < 0.05.

## RESULTS

The yield of the dry propolis extract was 41.43%. The green propolis sample contained a 0.95 ±0.44% flavonoid grade. Table 1 presents the histopathological data for the lingual epithelial lining in animals.

**Table 1:** Analysis of mean scores of epithelial histological changes and the classification according to the WHO and the binary system

GROUPS	Epithelial histological changes			Classification of epithelial changes	
	Architectural (Mean ±SD)	Cytological (Mean ±SD)	Affected epithelial layers	Binary system	WHO system
CTR1	1.5 ±0.22 a	0 a	Basal	BR	ND
CTR2	1.75 ±1.50 a	0 a	Basal	BR	ND
TUM1	4.83 ±0.40 b	2.33 ±1.36 b	Middle squamous	AR	DM
TUM2	4.50 ±1.51 b	3.16 ±2.13 b	Middle squamous	AR	DM
PROP1	4.0 ±1.22 b	0.80 ±0.30 a	Basal/Parabasal	AR	DL
PROP2	2.66 ±1.03 a	0.33 ±0.51 a	Basal/Parabasal	BR	DL*
PROP3	2.0 ±0.63 a	0.50 ±0.22 a	Basal/Parabasal	BR	DL*

SD - standard deviation

Epithelium with non-dysplasic alterations (ND); mild dysplasia (DL); moderate dysplasia (DM); severe dysplasia (DI); low risk (BR); high risk (AR);

Different letters in the same column are statistically different values (*p*<0.05).

(*) only in focal areas.

**Figure 1:**
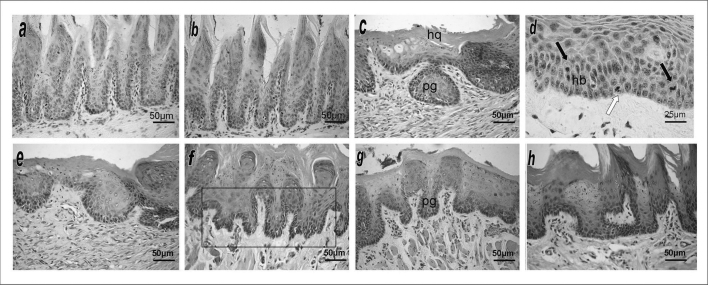
HE stained histological sections of the mucosal lining epithelium of the border of the tongue in experimental animals. (a) and (b) Negative control groups (CTR1 and CTR2) showing a normal epithelium. (c) Group TUM1 showing a hyperkeratinized epithelium (HQ) with dysplasia; note the droplet-shaped entrapped papillae (pg). (d) Group TUM1 showing moderate cell pleomorphism and hyperchromatic vell nuclei, evident basilar hyperplasia (hp) and basal mitosis (light arrow) and suprabasal mitosis (dark arrows). (e) Group TUM2 showing epithelium with dysplasia extending to the middle third, similar to TUM1. (f) Group PROP1 showing nuclear hyperchromatism in epithelial cells, highlighting droplet-shaped irregular papillae (Frame). (g) Group PROP2 showing rare and focal atypical findings expressed as discreet droplet-shaped papillae (pg) and mild nuclear hyperchromatism. (h) Group PROP3 showing only mildly disarrayed architecture of epithelial papillae and mildly hyperchromatic cell nuclei.

Groups CTR1 (Fig. 1a) and CTR2 (Fig. 1b) had the lowest number of morpho-architectural alterations (respectively 1.5 ±0.22 and 1.75 ±1.50); when present, these were focal areas of a duplicated basal layer and mild entrapment and hyperplasia of epithelial papillae. No cytologic atypia was seen. Epithelial alterations were classified as low risk in the binary system^11^ and as non-dysplasic morphological changes in the WHO criteria.^4^

Groups TUM1 (Fig. 1c/d) and TUM2 (Fig. 1e) had the highest scores for morpho-architectural alterations (respectively 4.83 ±0.40 and 4.50 ±1.51) and for cytologic changes (respectively 2.33 ±1.36 and 3.16 ±2.13). Morpho-architectural alterations were basilar hyperplasia, entrapped papillary projections (droplet shaped), increased number of mitotic figures (two or more in each histological field at 200x magnification), some of these in high epithelial layers, and dyskeratosis. Cytologic alterations were hyperchromatic and mild to moderately pleomorphic cell nuclei, and occasional increase in the nucleus/cytoplasm ratio. These cytomorphological alterations were limited to basal, parabasal, and middle squamous layers, although they were present throughout the epithelial lining. Epithelial alterations were classified as high risk in the binary system, and as moderate dysplasia in the WHO system.^4^

The PROP1 group (Fig. 1f) presented a high mean number of morpho-architectural alterations (4.0 ±1.22), and a low number of cytologic changes (0.80 ±0.30). These morpho-architectural alterations were similar to those found in the groups TUM1 and TUM2, and were limited to the lower third of the epithelial tissue (basal/parabasal); the cytologic changes were mild hyperchromatism of basal and parabasal cells. Epithelial alterations were classified as high risk in the binary system^11^ and as mild dysplasia in the WHO system.^4^

Groups PROP2 (Fig. 1g) and PROP3 (Fig. 1h) had similar results, with few morpho-architectural alterations (respectively 2.66 ±1.03 and 2.0 ±0.63) and cytologic changes (0.33 ±0.51 and 0.50 ±0.22). Papillary entrapment (droplet shaped projections), basilar hyperplasia, and a higher number of mitoses were found; cytologic changes, when present, were a mild increase in the number of basal and parabasal nuclei. These few changes were always located in the basal/parabasal layers, and only in focal areas. They were classified as low risk in the binary system, and as mild dysplasia in the WHO system.^4^

The statistical analysis showed that groups TUM1, TUM2, and PROP1 had a medium number of statistically similar morpho-architectural alterations (*p*>0.05); however, all were significantly higher than the findings in groups CTR1 and CTR2 (*p*<0.01), and PROP2 and PROP3 (*p*<0.05). Furthermore, the latter groups did not differ among each other (*p*>0.05). The cytologic findings in groups TUM1 and TUM2 were similar (*p*>0.05), but were significantly higher compared to the other groups (*p*<0.01).

## DISCUSSION

Experimental models and in vitro methods^7^ for inducing chemical carcinogenesis in rodents^10^ have been conducted to test the chemotherapeutic effects of natural products, especially during the initial phases of malignancies.

A chemical carcinogen, 9,10-dimethyl-1,2-benzatracene (DMBA), was used in the present study to induce lesions; this substance has been widely used in studies including induction of chemical carcinogenesis, for instance, by Lima, Taveira et al.,^12^ Chen et al.,^13^ Barros et al.,^14^ and Wang et al.^15^ The choice of the tongue as an anatomical site for lesions is based on the high rate of oral squamous cell carcinoma in this area, and the aggressive biological behavior of this type of squamous cell carcinoma, because of extensive vascularization in the tongue, according to Neville et al.,^3^ Bsoul et al.,^16^ and the INCA.^1^

The use of propolis with DMBA in the experimental group may be justified by the wide spectrum of biological properties that this phytotherapic agent possesses; it is an antimicrobial, antimycotic, immunomodulatory, healing, antioxidant, and antitumor agent, properties attributed to the presence of flavonoids in its composition.^17^ Flavonoids are phenolic compounds containing a hydroxyl radical directly bound to an aromatic ring. They are antibacterial because it can inhibit bacterial RNA-polymerase; they are immunomodulatory, antioxidants, and healing agents because of the ability to sequester or inhibit free radical formation.^9^, ^18^ In the present study, the green propolis sample that was used had a good amount of flavonoids (0.95 ±0.44%); the minimum amount is 0.25% (m/m), as established by the legislation on flavonoid compounds.^19^ Additionally, the yield of the dry extract (41.43%) was higher than the minimum specified value by the Ministry of Agriculture^19^ (11% m/v); it was, therefore, considered fully satisfactory. It should be said that other active compounds may be found in propolis, such as caffeic acid and its derivatives, which are also immunomodulatory and protective for the liver.^18^, ^20^

The negative control groups CTR1 and CTR2 in this study presented normal appearing oral mucosa with a paucity of morpho-structural alterations, expressed as focal areas of duplication of the basal layer and irregular papillae. Neville et al.^3^ and Regeziet al.,^21^ have suggested that architectural and cytologic alterations are common in areas subjected to frequent friction, as is the case for the dorsum of the tongue - an area of masticatory mucosa. These findings, therefore, may be interpreted as a reaction to trauma, and should not be mistaken for dysplasia.

The positive control groups TUM1 and TUM2 presented a significant quantity of architectural and cytologic changes that extended to the middle epithelial third. These findings point to the carcinogenic potential attributed to DMBA, according to the work of Lima & Taveira et al.,^12^ Chen et al.,^13^ Barros et al.,^14^ and Wang et al.^15^ It is important to note that endogenous factors, such as the site of the tumor to be induced, the species or lineage of the animal, and its health status, as well as exogenous factors, such as the diet and conditioning of animals, and further, the concentration, dilution vehicle and administration form of DMBA, may affect the time needed for the induction of malignant tumors by chemical carcinogenesis.

The experimental groups PROP1, PROP2, and PROP3 presented areas of morphological atypia limited to the lower epithelial thirds. Atypia here was diffusely distributed in the oral mucosa of group PROP1, whereas it was focally distributed in the lingual lining epithelium in groups PROP2 and PROP3. These findings suggest that administration of hydroalcoholic green propolis extracts decreased the dysplasia that had been induced by chemical carcinogenesis, and that chemoprotection was likely to be related with the dose of propolis. These findings appear to confirm the antitumor properties of this compounds, as previously reported by Luo et al.,^7^ Orsolic et al.,^6^ and Khalil.^5^

Veronez^22^ has suggested that caffeic acid phenethyl ester (CAFE) is the main substance with antitumor activity; it has shown cytotoxic activity against tumor cells, inhibiting oxidative processes essential for generating tumors, suppressing the oxidative destruction of polymorphonuclear leukocytes, and inhibiting protein, DNA, and RNA synthesis in tumor cells. It should be noted that there were no atypical mitoses or mitoses in epithelial parabasal and squamous layers in groups PROP2 and PROP3; these histological findings corroborate the mitosis-suppressing effect of CAFE.^22^

The WHO^4^ and binary system^1^ classifications for histological grading of oral mucosa epithelial dysplasias diverged in group PROP1, as some lesions were classified respectively as mild dysplasia and high risk dysplasia. Additionally, the statistical analysis showed that according to Kujan et al.'s^11^ binary system, there were no significant differences between the positive control groups (TUM1 and TUM2) and the group treated with the lowers concentration of propolis (PROP1). This suggests that the WHO^4^ classification is more reliable as it takes into account the architectural and cytologic changes across the full thickness of the oral mucosa, while the binary system^11^ appears to overestimate the grade of cytomorphological changes by considering only the quantity of architectural and cytologic changes found in the histopathological analysis but leaving aside the level of dysplasic involvement of the oral mucosa. The data also suggests that both systems yield similar results when morphological and structural changes are abundant, but that the binary system^11^ tends to overestimate the severity of cytological atypia when those changes are sparse or focal, resulting in erroneous diagnoses. It is also important to highlight the practical nature of the WHO^4^ classification compared to the binary system,^11^ which requires a more detailed analysis that is not practical in laboratory routines.

These results suggest a possible chemoprevention and antitumor activity of green propolis. However, further studies are needed to clarify the mechanism of these agents against oral chemically induced carcinogenesis.

## CONCLUSION

In the present study, DMBA effectively promoted chemical carcinogenesis by inducing dysplasia in the oral mucosa. It may be suggested that green propolis has a protective role during the process of chemically induced carcinogenesis on the tongue, and that this protection was directly related with its concentration in the hydroalcoholic extracts given by gavage. The WHO system for histologically grading epithelial dysplasia in the mouth was more practical and reliable for measuring the severity of epithelial dysplasia, compared to the binary system.

## ACKNOWLEDGEMENTS

The authors wish to acknowledge the Fundaçã o de Apoio a Pesquisa e Inovaçã o Tecnológica do Estado de Sergipe (FAPITEC) for funding for this study.

## References

[1] Inca - Instituto Nacional do Câncer (2009). Estimativa.

[2] Coletta RD, Graner E, Lopes MA, Vargas PA, Jorge JJR, Almeida OP. (2002). Os avanços da biologia molecular e o câncer bucal. Rev APCD.

[3] Neville BW, Damm DD, Allen CM, Bouquot JE. (2008). Patologia oral e maxilofacial.

[4] Barnes L, Evenson JW, Reichart P, Sindransky D. (2005). Pathology and genetics of head and neck tumours.

[5] Khalil ML. (2006). Biological activity of bee propolis in health and disease. Asian Pac J Cancer Prev.

[6] Orsolic N, Knezevic AH, Sver L, Terzic S, Basic I. (2004). Imunomodulatory and antimetastatic action of propolis and related polyphenolic compounds. J Ethnopharmacol.

[7] Luo J, Soh JW, Xing WQ, Mao Y, Matsuno T, Weinstein IB. (2001). PM-3 a benzo-gamma-pyran derivative isolated from propolis, inhibits grow of MCF-7 human breast cancer cells. Anticancer Res.

[8] Park YK, Ikegakl M, Abreu JAS, Alcici NMF. (1998). Estudo da preparaçã o dos extratos de Própolis e suas aplicaçõ es. Ciênc Tecnol Aliment.

[9] Adelmann J. (2005). Própolis: variabilidade composicional, correlaçã o com a flora e bioatividade antimicrobiana/antioxidante.

[10] Kavitha K, Manoharan S. (2006). Anticarcinogenic and antilipidperoxidative effects of tephrosia purpurea (linn.) Pers. In 7,12-dimethylbenz(a) anthracene (dmba) induced hamsters buccal pouch carcinoma. Indian J Pharmacol.

[11] Kujan O, Oliver RJ, Khattab A, Roberts SA, Thakker N, Sloan P. (2006). Evaluation of a new binary system of grading oral epithelial dysplasia for prediction of malignant transformation. Oral Oncol.

[12] Lima N L, Taveira LAA. (1999). Estudo das alteraçõ es morfológicas causadas pela induçã o concomitante de DMBA e bebidas alcoólicas de alto teor na carcinogênese química bucal. Rev FOB.

[13] Chen YK, Hsue SS, Lin LM. (2003). Correlation between inducible nitric oxide synthase and p53 expression for DMBA-induced hamster bucal-pouch carcinomas. Oral Dis.

[14] Barros ACSD, Muranaka ENK, Mori LP, Pelizon CHT, Iriya K, Giocondo G (2004). Induçã o da carcinogênese mamária experimental em ratas com 7,12 - dimetil-benz(a)antraceno. Rev Hosp Clín Fac Med S Paulo.

[15] Wang WC, Liang SL, Chen YK, Lin LM. (2008). The therapeutic effect of fractionated radiation on DMBA-induced hamster buccal pouch squamous cell carcinomas. Oral Oncol.

[16] Bsoul SA, Huber AM, Terezhaimy GT. (2005). Squamaus Cell Carcinoma of the oral tissues: A comprehensive review for oral healthcare providers. J Contemp Dent Pract.

[17] Daugsh A, Moraes CS, Fort P, Park YK (2007). Brazilian red própolis-chemical composition and botanical origin. ECAM.

[18] Bosio K, Avanzin C, D'Avolio A, Ozino O, Savoia D (2000). In vitro activity of própolis against Streptococcus pyogenes. Microbiology.

[19] Brasil. Ministério da Agricultura, Pecuária e do Abastecimento. Instruçã o Normativa n^o^ 3, de 19 de janeiro de 2001. Aprova os regulamentos Técnicos de Identidade e Qualidade de Apitoxina, Cera de Abelha, Geléia Real, Geléia Real Liofilizada, Pólen Apícola, Própolis e Extrato de Própolis, conforme consta dos Anexos desta Instruçã o Normativa. Publicado no Diário Oficial da União de 23/01/2001, Seçã o 1, Página 18.

[20] Castaldo S, Capasso F. (2002). Própolis, an old remedy used in modern medicine. Fitoterapia.

[21] Regezi JA, Sciuba JJ, Jordan RCK. (2008). Patologia bucal: correlaçõ es clinicopatológicas.

[22] Veronez R. (2000). Revisão de literatura: própolis na clínica médica internacional.

